# The impact of socio-economic disadvantage on rates of hospital separations for diabetes-related foot disease in Victoria, Australia

**DOI:** 10.1186/1757-1146-4-17

**Published:** 2011-06-20

**Authors:** Shan M Bergin, Caroline A Brand, Peter G Colman, Don A Campbell

**Affiliations:** 1 Podiatry Department, Dandenong Hospital, Melbourne, Victoria, 3172, Australia; 2 Clinical Epidemiology and Health Service Evaluation Unit, Royal Melbourne Hospital, Melbourne, Victoria, 3052, Australia; 3 Department of Diabetes and Endocrinology, Royal Melbourne Hospital, Melbourne, Victoria, 3052, Australia; 4 Department of General Medicine, Monash University, Melbourne, Victoria, 3169, Australia

## Abstract

**Background:**

Information describing variation in health outcomes for individuals with diabetes related foot disease, across socioeconomic strata is lacking. The aim of this study was to investigate variation in rates of hospital separations for diabetes related foot disease and the relationship with levels of social advantage and disadvantage.

**Methods:**

Using the Index of Relative Socioeconomic Disadvantage (IRSD) each local government area (LGA) across Victoria was ranked from most to least disadvantaged. Those LGAs ranked at the lowest end of the scale and therefore at greater disadvantage (Group D) were compared with those at the highest end of the scale (Group A), in terms of total and per capita hospital separations for peripheral neuropathy, peripheral vascular disease, foot ulceration, cellulitis and osteomyelitis and amputation. Hospital separations data were compiled from the Victorian Admitted Episodes Database.

**Results:**

Total and per capita separations were 2,268 (75.3/1,000 with diabetes) and 2,734 (62.3/1,000 with diabetes) for Group D and Group A respectively. Most notable variation was for foot ulceration (Group D, 18.1/1,000 *versus *Group A, 12.7/1,000, rate ratio 1.4, 95% CI 1.3, 1.6) and below knee amputation (Group D 7.4/1,000 *versus *Group A 4.1/1,000, rate ratio 1.8, 95% CI 1.5, 2.2). Males recorded a greater overall number of hospital separations across both socioeconomic strata with 66.2% of all separations for Group D and 81.0% of all separations for Group A recorded by males. However, when comparing mean age, males from Group D tended to be younger compared with males from Group A (mean age; 53.0 years *versus *68.7 years).

**Conclusion:**

Variation appears to exist for hospital separations for diabetes related foot disease across socioeconomic strata. Specific strategies should be incorporated into health policy and planning to combat disparities between health outcomes and social status.

## Background

Inequalities in the overall burden of chronic disease across socioeconomic strata are well documented [1,2]. For those in lower socio-economic strata, disparities exist for both overall disease prevalence and health care outcomes. Further to this, there are recognised inequalities in access to health care, as well as documented increased mortality and morbidity rates in less advantaged communities [3-6].

Information about socio-economic disparities, especially when linked to inequalities in health outcomes, can impact on health care planning and policy. In particular, it can inform decisions about appropriate allocation of resources. Some health conditions, such as cardiovascular disease and some cancers have been well characterised according to social determinants in selected Australian populations [7,8]. However, there remain some chronic conditions that are yet to be fully explored in terms of disparities in disease prevalence across different communities.

Diabetes related foot disease including peripheral neuropathy, peripheral vascular disease, ulceration and amputation, contribute significantly to the overall burden of disease in Australia [9,10]. However, prevalence rates for diabetes related foot disease have yet to be quantified according to socio-economic status. Furthermore there is little evidence about geographical variation in social determinants and the relationship with health outcomes for people with these common disorders in Australia.

Determination of any relationship between variables such as socioeconomic status and health outcomes becomes increasingly important when chronic disease becomes particularly complex, as is the case with diabetes related foot disease, and the care required is provided via acute and community based health care settings. Socioeconomic status in Australian populations is determined using Census of Population and Housing data (referred to as 'census' here) collected by the Australian Bureau of Statistics every five years [11]. As an overall indication of relative advantage and/or disadvantage across small geographic areas, information such as household income, level of education and levels of unemployment, are used to assign Socioeconomic Indexes for Areas (SEIFA) [12]. The aim of this study was to investigate the relationship between geographical variation in hospital separations for diabetes related foot disease and socioeconomic status.

## Methods

This study was approved by The Melbourne Health Human Research and Ethics Committee, The Monash University Standing Committee on Ethics for Research Involving Humans and The Department of Human Services Victoria Human Research Ethics Committee.

### Socioeconomic Indexes for Areas (SEIFAs)

Using Department of Human Services Victoria statewide maps, each LGA of Victoria was identified. A LGA is defined as an undivided geographical area that is the responsibility of a single local government [13]. Each LGA is comprised of one or more, smaller geographic areas known as Statistical Local Areas. Regions or Statistical Local Areas incorporated into a single LGA may change over time and boundaries that define each LGA may also shift.

Subsequent to the mapping of each Victorian LGA, all postcodes that fell within each individual LGA were identified using the Australia Post Postcode Datafile and all corresponding SEIFAs for year 2006, allocated by the Australian Bureau of Statistics, were identified [14]. There are four indexes that are used to determine SEIFA and each uses different data that is collected and analysed subsequent to each 5 yearly government census [12]. For the purposes of this study, we have used the IRSD, where an index or decile of 1 indicates those areas in the bottom 10% of the state, reflecting those areas at most disadvantage. A decile of 10 indicates those areas in the top 10% of the state which are areas of least disadvantage.

In order to allocate a rank under IRSD, the Australian Bureau of Statistics analyses 17 different census variables, including proportion of low income households per area, proportion of residents who don't speak English well and proportion of people per area with no post-school qualifications. It should be noted that each SEIFA applied is a summary index for a total area, in this case an LGA, and is not an indication of the level of advantage or disadvantage for each individual within that area. Once each LGA had been ranked according to the 2006 IRSD allocation, all LGAs with an index of 1 or 2 (most disadvantaged) and those with an index of 9 or 10 (most advantaged) were identified and their corresponding postcodes recorded.

### Hospital separations

A series of International Classification of Diseases (ICD) codes were identified that describe diabetes related peripheral neuropathy, peripheral vascular disease, foot ulceration, infection (soft tissue and bone) and amputation (above and below knee). Fourteen of the identified ICD codes were used to interrogate the Victorian Admitted Episodes Database (VAED) for all hospital separations occurring for years 2004/05 and 2005/06 (Table 1). Where the ICD codes were not specific to diabetes (eg. E1073, type 1 diabetes with foot ulcer), separations were only captured if the individual recorded the code of interest (eg. L0302, toe cellulitis) and the codes for type 1 or type 2 diabetes.

**Table 1:** International Classification of Diseases (ICD) codes identified from ICD 10-AM version 4, used to interrogate the Victorian Admitted Episodes Database for all hospital separations for specified local government areas for year 2005/06.

ICD codes	Definitions
E1051, E1052, E1151, E1152, E1451, E1452	Peripheral vascular disease for type 1, type 2 and unspecified diabetes with and without gangrene
E1073, E1173, E1473	Foot ulcer in type 1, type 2 and unspecified diabetes
L0302	Toe cellulitis
M8697	Osteomyelitis (unspecified)
Z894	Foot amputation
Z895	Below knee amputation
Z896	Above knee amputation

The VAED held by The Department of Human Services, Victoria, includes morbidity data on all individuals accessing acute health care within the public, private and rehabilitation health care settings across Victoria [15]. The VAED records and reports on all hospital separations for all admissions. A hospital separation is defined as 'an episode of care' provided during a single hospital admission, therefore, one patient may record multiple hospital separations during a single admission.

For the purposes of this study, separations recorded during 2005/06 for ICD codes reflecting peripheral vascular disease, foot ulceration, toe cellulitis, osteomyelitis (unspecified) and amputation (including foot amputation, below and above knee amputation) were analysed for all LGAs identified as having an IRSD of 1, 2, 9 or 10. Hospital separations and LGAs were matched using postcode data collected from the VAED and LGA postcodes determined via the Australia Post Postcode Datafile. Demographic data, including age, gender were also collected.

### Additional data

Census data from 2006 was used to determine total population per included LGA and 2006 total population and percentage population with diabetes was determined for each area using data from Diabetes Australia (Victoria) [16]. Diabetes prevalence data was calculated using 2006 census data and registration numbers from the National Diabetes Services Scheme; a government initiative that provides products such as syringes and blood glucose testing equipment, at a subsidised rate. Prevalence data was calculated using a total population estimate generated for each LGA using Australian Bureau of Statistics five year growth rates for years 2001-2005. Using the 2006 population estimate, Diabetes Australia (Victoria) then calculated a percentage estimate for diabetes prevalence per LGA, by dividing the number of people registered with The National Diabetes Services Scheme by the estimated population for that LGA.

### Statistical analysis

All data collected for those LGAs with an IRSD of 1 or 2 was analysed together (Group D) as was all data collected for those LGAs with an IRSD of 9 or 10 (Group A). Separations data for each cluster of LGAs was analysed as overall frequencies and are reported as total separations overall and total separations per ICD code. Where multiple ICD codes were used to extract data relating to a single diabetes related foot disease (eg. peripheral vascular disease), all data was combined for ease of analysis. Separations data is also reported as per capita separations/1,000 total population with diabetes per LGA cluster. Mean age and male/female (%) data is also reported for all separations.

A crude rate ratio was calculated for all per capita data and is reported as rate ratio estimate per ICD code with 95% confidence interval (CI). This rate ratio was unadjusted for age and sex as the data required to account for these possible confounders during analysis was unavailable. Effect estimates for age were calculated using the unpaired t-test and are reported as mean difference with 95% CI. Percentage differences for gender were analysed using chi-square and are reported as odds ratios with 95% CI.

## Results

From 79 LGAs across Victoria, 16 were identified as having an IRSD of 1 (n = 8) or 2 (n = 8) and 16 as having an IRSD of 9 (n = 8) or 10 (n = 8). Total population across Group D was 798,007 of which, 42% were male and 44% of the total population were over the age of 45 years. This compares to an overall population of 1,584,898 in Group A; a difference of 786,891 people. Within Group A, 49% of the population were male and 39% of the population were over the age of 45 years. Total population with diabetes for Group D was 30,110 (3.8% of total population) compared with 43,904 (2.8% of total population) for Group A. Descriptive data for all included LGAs can be seen in Table 2.

**Table 2:** Descriptive data for all included Local Government Areas (LGAs).

LGA	IRSD	Total population	Population with diabetes mellitus	% population with diabetes mellitus
Loddon	1	8,095	708	8.5
Central Goldfields	1	12,739	460	3.5
Northern Grampians	1	12,330	683	5.4
Pyrenees	1	6,772	539	8.3
La Trobe	1	72,075	2,275	3.3
Brimbank	1	174,746	8,143	4.6
Maribyrnong	1	66,145	2,267	3.7
Greater Dandenong	1	130,751	5,089	4.0
Mildura	2	51,824	851	1.6
Swan Hill	2	21,285	566	2.6
Hindmarsh	2	6,235	271	4.3
Yarriambiack	2	7,742	376	4.8
Ararat	2	11,653	487	4.3
Glenelg	2	20,525	1,043	5.2
East Gippsland	2	41,361	1,991	4.8
Hume	2	153,729	4,361	2.8

**TOTAL**	16	798,007	30,110	3.8

Macedon Ranges	9	39,989	1,013	2.4
Queenscliffe	9	3,150	25	0.8
Banyule	9	119,347	3,115	2.7
Melbourne	9	76,678	1,670	2.4
Knox	9	152,388	4,110	2.7
Maroondah	9	102,478	2,966	3.0
Monash	9	169,829	5,825	3.6
Whitehorse	9	151,223	5,049	3.5
Surf Coast	10	22,802	1,414	6.0
Nilumbik	10	62,022	1,168	1.9
Manningham	10	115,702	3,627	3.2
Booroondarra	10	162,285	3,654	2.3
Stonnington	10	95,235	2,004	2.2
Bayside	10	91,726	2,313	2.6
Glen Eira	10	129,576	4,164	3.4
Port Phillip	10	90,458	1,787	2.1

**TOTAL**	16	1,584,898	43,904	2.8

2006 census data was used to determine total population per LGA. Total and percentage population with diabetes per LGA was calculated using census population figures and registration numbers from the National Diabetes Services Scheme. Index of Relative Socioeconomic Disadvantage (IRSD) rankings were sourced from the Australian Bureau of Statistics (2006).

Summary data, for total and per capita separations for each LGA cluster can be seen in Table 3.

**Table 3:** Combined summary data for hospital separations according to International Classification of Diseases code and Local Government Areas (LGA) cluster.

	Peripheral vascular disease	Ulcer	Cellulitis	Osteomyelitis	Foot amputation	Below knee amputation	Above knee amputation
Total separations							
Group D	972	546	129	162	163	223	73
Group A	1238	556	152	228	301	180	79
Per capita separations							
Group D	32.2	18.1	4.3	5.4	5.4	7.4	2.4
Group A	28.2	12.7	3.4	5.2	6.9	4.1	1.8
Rate ratio (95% CI)	1.15 (1.1, 1.3)	1.4 (1.3, 1.6)	1.24 (0.1, 1.6)	1.04 (0.8, 1.3)	0.8 (0.7, 1.0)	1.8 (1.5, 2.2)	1.35 (0.1, 1.9)

Mean age (years)							
Males							
Group D	71.5	66.8	52.5	62.0	64.0	72.3	53.8
Group A	70.0	69.0	69.7	69.8	69.0	70.6	62.7
Mean difference (95% CI)	1.5 (0.9, 2.0)	-2.2 (-3.2, -1.2)	-17.2 (-20, -14)	-7.8 (-11, -5.4)	-7.0 (-9, -4)	1.7 (0.2, 3.2)	-8.9 (-13, -4.5)
Females							
Group D	75.4	57.5	57.2	71.5	69.2	75.8	76.7
Group A	74.6	76.0	69.7	80.0	72.2	68.7	73.9
Mean difference (95% CI)	0.8 (-0.1, 1.7)	-18.5 (-20, -17)	-12.5 (-16, -9.1)	-8.5 (-12, -5.4)	-3.0 (-7, -0.9)	7.1 (2.0, 12.2)	2.8 (-1.4, 7.0)

Gender (%)							
Males							
Group D	68.8	65.6	58.0	45.0	77.0	74.0	51.0
Group A	60.0	54.8	69.0	53.5	61.5	71.0	64.5
Females							
Group D	32.0	34.4	42.0	55.0	23.0	26.0	49.0
Group A	40.0	45.2	31.0	46.5	38.5	29.0	35.5
Odds ratio (95% CI)	1.4 (1.2, 1.7)	1.6 (1.2, 2.0)	0.62 (0.4, 1.0)	0.71 (0.5, 1.1)	2.1 (1.3, 3.2)	1.0 (0.6, 1.5)	0.57 (0.3, 1.1)

Total separations are reported as absolute frequencies and per capita data refers to number of separations per 1,000 total population with diabetes per LGA cluster. Rate ratios are unadjusted for age and sex as insufficient data was available for this type of analysis. Effect estimates for age were calculated using unpaired t-test and are reported as mean difference and percentage differences for gender were analysed using chi-square and are reported as odds ratios.

Total separations overall for LGAs within Group D was 2,268, which equates to 75.3 separations/1,000 people with diabetes. From this group, 66.2% of all separations were recorded by males with a mean age of 53 years. For all hospital separations recorded by females from this LGA cluster the mean age was 69 years.

For those areas within Group A total separations overall was 2,734 or 62.3/1,000 people with diabetes. Of these, 81% were recorded by males with a mean age of 68.7 years. Females from within the same cluster had a mean age of 73.6 years.

Per capita separations were higher for 5 out of 7 components of diabetes related foot disease evaluated for Group D. The greatest differences in per capita separations were seen for foot ulcer (18.1/1,000 with diabetes *versus *12.7/1,000 with diabetes, rate ratio 1.4 [1.3, 1.6]), and below knee amputation (7.4/1,000 with diabetes *versus *4.1/1,000 with diabetes, rate ratio 1.8 [1.5, 2.2]). This equates to a 40% increased rate of hospital separations for foot ulcer and an even more significant increased rate of separations for below knee amputation for those individuals residing in less advantaged areas of the state. Those areas within Group A recorded a higher per capita rate of separations for foot amputation (6.9/1,000 with diabetes *versus *5.4/1,000 with diabetes, rate ratio 0.8, [0.7, 1.0]) when compared to those LGAs with a lower ranking.

Significant associations were found between gender and all components of diabetes related foot disease analysed except for below knee amputation, with a greater percentage of males from LGAs within Group D likely to record hospital separations. The greatest significance was found for PVD (OR 1.4 [1.2, 1.7]), foot ulcer (OR 1.6 [1.2, 2.0] and foot amputation (OR 2.1 [1.3, 3.2]).

Age was also a significant factor with both males and females from Group D likely to be younger at the time the hospital separation was recorded, when compared to their counterparts from more advantaged areas of the state. This was particularly true for cellulitis (mean difference -17.2 years [-20.0, -14.0] and above knee amputation (mean difference -8.9 years [-13, -4.5]) for separations recorded by males and foot ulcer (mean difference -18.5 years [-20.0, -17.0]) and cellulitis (mean difference -12.5 [-16.0, -9.1]) for separations recorded by females.

## Discussion

The findings of this study indicate there is variation between total hospital separations for diabetes related foot disease across socioeconomic strata in Victoria. Those LGAs with an IRSD of 1 or 2 recorded a greater number of overall per capita separations for diabetes related foot disease and recorded a greater number of per capita separations for 5 out of 7 of the individual components of diabetes related foot disease evaluated. Males recorded a greater number of hospital separations compared to females across both LGA clusters, however both males and females from more disadvantaged areas of the state, were likely to be younger at the time the hospital separation was recorded, when compared with their counterparts from areas with greater relative advantage.

The findings from this study, believed to be the first of its kind in Australia, have implications for the distribution of required health care services for management of diabetes related foot disease across Victoria. Whilst it is recognised that other factors such as compliance may play a role in the development of diabetes related complications, including foot disorders, it is also important that disparities in access to health care do not contribute to increased complication rates in disadvantaged areas. Although we have been unable to find any published studies reporting on hospital separations or differences in prevalence or incidence for diabetes related foot disease across SEIFA within Australian populations, a limited number of international studies have demonstrated a relationship between socioeconomic determinants and rates of diabetes related foot disease.

A study by Weng et al [17] conducted in the UK investigated 610 patients with diabetes attending an inner city hospital for the first time, and found that those individuals living in areas classified as 'deprived' were 3.5 times more likely to experience foot ulceration or amputation compared to individuals living in areas classed as 'intermediate', and were twice as likely to experience these complications compared to those living in more 'prosperous' areas. Bihan et al [18] conducted a cross sectional prevalence study that included 135 patients with diabetes admitted to a French hospital. Deprivation (this study used individual deprivation scores as opposed to measures for area deprivation) was assessed in correlation with the prevalence of identified diabetes complications. This study found that patients classed as socioeconomically 'deprived' were significantly more likely to experience microvascular complications such as peripheral neuropathy, when compared to those from less deprived areas. Studies from the USA have also reported positive associations between increased overall morbidity and mortality and socioeconomic disadvantage in individuals with diabetes [19,20].

The findings from this study provide important data about the relationship between socioeconomic status, hospital separations and diabetes related foot disease that was previously lacking. However, it must be acknowledged that hospital separations data may potentially over or even underestimate the true number of hospital based episodes of care provided for diabetes related foot disease; this phenomenon is a function of current coding principles and the methodologies used to collect these health care indicators are subject to human error and variations in the interpretation of medical record information [10]. However, there is some evidence to suggest accuracy of coding is sufficient to make reliable estimates regarding both hospital admissions and hospital separations with audits around accuracy of data collected via the VAED supporting the usefulness of this type of retrospective data collection [21].

Diabetes prevalence rates used for this study may also be underestimated due to the methodology used by Diabetes Australia (Victoria) to calculate small area data. Not all individuals with diabetes register with the National Diabetes Services Scheme, and some, such as indigenous Australians are unlikely to be represented. This may mean that the disparities identified here between hospital separations for diabetes related foot disease and socioeconomic status may in fact be greater than first thought.

## Conclusion

This paper has demonstrated that rates of hospital separations for diabetes related foot disease are probably increased in areas that are socioeconomically disadvantaged. All attempts should be made to ensure coding data is as accurate as possible and this data should then be captured across wider populations with diabetes related foot disease within Australia, and be utilised to plan and resource health care services accordingly. Disparities in access to, and utilisation of, required health care services should be minimised in order to ensure clinical outcomes are not determined by socioeconomic status.

## List of abbreviations

ICD: International Classification of Diseases; LGA: Local Government Area; IRSD: Index of Relative Socioeconomic Disadvantage; SEIFA: Socioeconomic Indexes for Areas; VAED: Victorian Admitted Episodes Database.

## Competing interests

The authors declare that they have no competing interests.

## Authors' contributions

SB conceived of the study, designed the study methodology, collected and analysed data and drafted the manuscript. CB advised on study methodology and provided editorial support for the manuscript. PC advised on study methodology and provided editorial support for the manuscript. DC advised on study methodology, data analysis and provided editorial support for the manuscript. All authors read and approved the final manuscript.

## References

[B1] Mete C Cioffi JP Lichtveld M Are public health services available where they are most needed? An examination of local health department services J Public Health Manag Pr 2003 9 214 223 10.1097/00124784-200305000-00006 12747318

[B2] Singh-Manoux A Adler NE Marmot MG Subjective social status: its determinants and it association with measures of ill-health in the Whitehall II study Soc Sci Med 2003 56 1321 1333 10.1016/S0277-9536(02)00131-4 12600368

[B3] van Doorslaer E Koolman X Explaining the differences in income-related health inequalities across European countries Health Econ 2004 13 609 628 10.1002/hec.918 15259042

[B4] Makinen M Waters H Rauch M Almagambetova N Bitran R Gilson L McIntyre D Pannarunothai S Prieto AL Ubilla G Ram S Inequalities in health care use and expenditure: empirical data from eight developing countries and countries in transition Bull World Health Organ 2000 78 55 65 10686733 PMC2560608

[B5] Walker B Figgs LW Zahm SH Differences in cancer incidence, mortality and survival between African Americans and whites Environ Health Perspect 1995 103 Suppl 8 S275 S281 10.1289/ehp.95103s8275 PMC1518956 8741798

[B6] Mackenbach JP Stirbu I Roskam AJ Schaap MM Menvielle G Leinsalu M Kunst AE European Union Working Group on Socioeconomic Inequalities in Health Socioeconomic inequalities in health in 22 European countries New Eng J Med 2008 358 2468 2481 10.1056/NEJMsa0707519 18525043

[B7] Thomas DP Condon JR Anderson IP Li SQ Halpin S Cunningham J Guthridge SL Long-term trends in indigenous deaths from chronic diseases in the Northern Territory: a foot on the brake, a foot on the accelerator Med J Aust 2006 185 145 149 16893354 10.5694/j.1326-5377.2006.tb00501.x

[B8] Cunningham J Rumbold AR Zhang X Condon JR Incidence, aetiology and outcomes of cancer in indigenous peoples in Australia Lancet Oncol 2008 9 585 595 10.1016/S1470-2045(08)70150-5 18510990

[B9] Lawrence SM Wraight PR Campbell DA Colman PG Assessment and management of acute diabetes related foot complications: room for improvement Inter Med J 2004 34 229 233 10.1111/j.1444-0903.2004.00590.x 15151667

[B10] Australian Institute of Health and Welfare Diabetes hospitalisations in Australia, 2003-04 2006 Canberra: Australian Institute of Health and Welfare Bulletin no 47. Cat. No. 84.

[B11] How Australia takes a census http://www.abs.gov.au/AUSSTATS/abs@.nsf/DetailsPage/2903.02006

[B12] Socio-economic Indexes for Areas - Technical Paper http://www.abs.gov.au/websitedbs/D3310114.nsf/home/Seifa_entry_page

[B13] Australian Standard Geographical Classification (ASGC) http://www.abs.gov.au/AUSSTATS/abs@.nsf/DetailsPage/1216.02004?OpenDocument 10.1111/j.1444-0938.2011.00590.x 21426397

[B14] Australia Post Postcode Datafile http://www.austpost.com.au/

[B15] Department of Human Services, Victoria. Victorian Admitted Episodes Database User Manual 18^th ^Ed. (2008/09) http://www.health.vic.gov.au/hdss/vaed/index.htm

[B16] The Australian Bureau of Statistics. 2006 census data by location http://www.abs.gov.au/

[B17] Weng C Coppini DV Sönksen PH Geographic and social factors are related to increased morbidity and mortality rates in diabetic patients Diabet Med 2000 17 612 617 10.1046/j.1464-5491.2000.00352.x 11073184

[B18] Bihan H Laurent S Sass C Nguyen G Huot C Moulin JJ Guegen R Le Toumelin P Le Clésiau H La Rosa E Reach G Cohen R Association among individual deprivation, glycaemic control, and diabetes complications: The EPICES score Diabetes Care 2005 28 2680 2685 10.2337/diacare.28.11.2680 16249539

[B19] Tseng CW Tierney EF Gerzoff RB Dudley RA Waitzfelder B Ackermann RT Karter AJ Piette J Crosson JC Ngo-Metzger Q Chung R Mangione CM Race/ethnicity and economic differences in cost-related medication underuse among insured adults with diabetes: the Translating Research into Action for Diabetes Study Diabetes Care 2008 31 261 266 18000177 10.2337/dc07-1341

[B20] Kirk JK D'Agostino RB Jr Bell RA Passmore LV Bonds DE Karter AJ Narayan KM Disparities in HbA1c levels between African-Americans and non-Hispanic white adults with diabetes: a meta-analysis Diabetes Care 2006 29 2130 2136 10.2337/dc05-1973 16936167 PMC3557948

[B21] Department of Human Services, Victoria. Audits of Hospital Admitted Patient Data 2005-09 http://www.health.vic.gov.au/hdss/vaed/audit/index.htm

